# Occlusion of the Ribosome Binding Site Connects the Translational Initiation Frequency, mRNA Stability and Premature Transcription Termination

**DOI:** 10.3389/fmicb.2017.00362

**Published:** 2017-03-14

**Authors:** Mette Eriksen, Kim Sneppen, Steen Pedersen, Namiko Mitarai

**Affiliations:** ^1^Department of Biology, University of CopenhagenCopenhagen, Denmark; ^2^Center for Models of Life, Niels Bohr Institute, University of CopenhagenCopenhagen, Denmark

**Keywords:** gene expression, translation initiation frequency, mRNA stability, transcription termination, modeling, occlusion time

## Abstract

Protein production is controlled by ribosome binding to the messenger RNA (mRNA), quantified in part by the binding affinity between the ribosome and the ribosome binding sequence on the mRNA. Using the *E. coli lac* operon as model, Ringquist et al. (1992) found a more than 1,000-fold difference in protein yield when varying the Shine-Dalgarno sequence and its distance to the translation start site. Their proposed model accounted for this large variation by only a variation in the binding affinity and the subsequent initiation rate. Here we demonstrate that the decrease in protein yield with weaker ribosome binding sites in addition is caused by a decreased mRNA stability, and by an increased rate of premature transcription termination. Using different ribosome binding site sequences of the *E. coli lacZ* gene, we found that an approximately 100-fold span in protein expression could be subdivided into three mechanisms that each affected expression 3- to 6-fold. Our experiments is consistent with a two-step ribosome initiation model, in which occlusion of the initial part of the mRNA by a ribosome simultaneously protects the mRNA from both premature transcription termination and degradation: The premature termination we suggest is coupled to the absence of occlusion that allows binding of transcription termination factor, possibly Rho. The mRNA stability is explained by occlusion that prevents binding of the degrading enzymes. In our proposed scenario, a mRNA with lower translation initiation rate would at the same time be “hit” by an increased premature termination and a shorter life-time. Our model further suggests that the transcription from most if not all natural promoters is substantially influenced by premature termination.

## Introduction

The protein yield per mRNA is traditionally thought to be a relatively simple function of ribosome binding to the ribosome binding site (RBS). The study of *lacZ* gene from *E. coli* (Ringquist et al., 1992) extended this view and quantified how the expression of a gene was affected by the Shine-Dalgarne (SD) sequence, its distance from the translation start, as well as of the first codons. In that study the produced β-galactosidase spanned more than thousand-fold. This huge range in protein yield indicated that the mRNA with the weak RBS were only rarely exposed to a ribosome. Given the current knowledge of coupling between the first ribosome and the RNA-polymerase (Burmann et al., 2010; Proshkin et al., 2010), one might question how the RNA-polymerase is able to transcribe such low yield mRNAs at all. In the study of Ringquist et al. only the β-galactosidase expression levels in the various *lacZ* variants were measured. These data are therefore not sufficient to determine whether an altered translation initiation rate is the only direct cause for the amount of expressed protein.

We have previously shown that the functional stability of an mRNA depends on the ribosome occupancy in the initial codon region, extending to include the first 50 codons on the mRNA. The mechanism for this, we envisaged, was that a lower ribosome occupancy on this part of the mRNA generated a larger target for degradation of the mRNA (Pedersen et al., 2011). It is therefore natural to suggest that the reported low expression for mRNA with weak RBS was also associated to the message stability. In this paper, we report the mRNA stabilities from a selected subset of the *lacZ* messages from the study of Ringquist et al. (1992).

In bacterial transcription, it is essential that a contact between the elongating RNA polymerase and the initial ribosome is established. If the contact for example is broken by a translational stop-codon, the transcription is terminated by transcription termination factor Rho (Beckwith, 1963; Newton et al., 1965). Altering the RBS will affect the time for the first ribosome to contact the RNA polymerase. For a weak RBS, it may take so long that the transcription terminates. We therefore also measured the premature transcription termination in these *lacZ* SD variants, and found a substantial termination for weak SD sequences.

## Materials and methods

The experiments were carried out in MC1000 (Casadaban and Cohen, 1980) and *recA*/F'*lacI*^*q1*^ *lacZ*::Tn*5*. We incubated cultures at 37°C and used the MOPS medium (Neidhardt et al., 1974) supplemented with 25 μg/ml L-leucine, 0.4% glycerol, 2.5 μg/ml thiamine and 100 μg/ml ampicillin.

To facilitate cloning of the altered RBS in the previous study (Ringquist et al., 1992), a change of the early *lacZ* codons was needed in order to generate a restriction enzyme site. Such change might alter the occlusion time (the time a preceding ribosome mask the RBS) of the ribosome binding site or the binding constant itself and thereby alter the initiation frequency. Thanks to the development of the recombineering technique (Sawitzke et al., 2007) one can now insert sequences at will. In order to compare the results both to the previous study and to the expression of the wild type, (wt) *lacZ* gene used in our study (Pedersen et al., 2011), we constructed seven *lacZ* variants: sd5-*lacZ-rq*, SD12-*lacZ-rq*, SD8-*lacZ-rq*, with the *lacZ* coding sequence used by Ringquist et al. (1992) and also sd5-*lacZ-wt*, SD12-*lacZ-wt*, SD8-*lacZ-wt*, and wt-SD-*lacZ-wt*. Throughout our paper we use the nomenclature introduced by Ringquist et al. (1992), where sd5 means the reasonably good SD sequence AAGGA located 5 nucleotides from the start codon, and SD8 and SD12 the very strong SD sequence TAAGGAGGA located 8 respectively 12 nucleotides from the start codon. The relevant part of these *lacZ* variants were sequenced and shown in Table 1.

**Table 1:** **Sequences of the *lacZ* variant mRNAs used**.

**Plasmid**	**SD sequence and *lacZ* sequence**
pMET1 stop codon in codon 3	CAATTTCACACAGGAAACAGCT ATG ACC **TAG** ATT ACG GAT^*^
wt-SD *lacZ-wt, lacYA*	CAATTTCACACAGGAAACAGCT ATG ACC ATG ATT ACG GAT^*^
sd5-SD *lacZ-rq, lacYA*	aaaaaaggaaaaaa ATG cag gat ccc GTC^$^ GTT
SD8-SD *lacZ-rq, lacYA*	aaaataaggaggaaaaaaaa ATG cag gat ccc GTC^$^ GTT
SD12-SD *lacZ-rq, lacYA*	taaggaggaaaaaaaaaaaa ATG cag gat ccc GTC^$^ GTT
sd5-SD *lacZ-wt, lacYA*	aaaaaaggaaaaaa ATG ACC ATG ATT ACG GAT^*^
SD8-SD *lacZ-wt*, lacYA	aaaataaggaggaaaaaaaa ATG ACC ATG ATT ACG GAT^*^
SD12-SD *lacZ-wt*, lacYA	taaggaggaaaaaaaaaaaa ATG ACC ATG ATT ACG GAT^*^

* *Designates codon six in the wt lacZ sequence. The sequence distal to codon six in these variants is the wt lacZ sequence*.

$ *Designates codon 10 in the wt lacZ sequence, which now is codon five in the lacZ-rq. The sequence distal to codon five in these variants is the wt lacZ sequence. Lower case font indicates where the sequences differ from the wt lacZ. The four, five, or nine bases complementary to the 3′ end of 16S RNA and the ATG start codon are underlined*.

All plasmids are derived from pMAS2, a derivative of pBR322 and carrying the wt *lacZ* (Sørensen et al., 1989). The variants in *lacZ* were made by recombineering (Sawitzke et al., 2007) in HME70 F'*lacI*^*q1*^*lacZ*::Tn*5* by first isolating a variant, pMET1 with a UAG stop codon in codon 3 of the *lacZ* gene (white on X-gal plates) into pMAS2. We then made variants with the desired sequences, screening for blue recombinants on X-gal plates.

We next reconstructed the complete *lac* operon in the above variants by cloning an *EcoRI-EcoRI* fragment (from the *EcoRI* site late in *lacZ* to the *EcoRI* site in the *cam* gene). This fragment contained *lacY* and *lacA* and came from pSB4027, that carried the complete *lac* operon on the vector pACYC184 (pSB4027 was provided by S. Brown).

The assays for LacZ and LacA enzyme activity were performed essentially as described (Miller, 1972), but without the inducer, IPTG in the overnight culture. The activity for both enzymes after induction were plotted vs. the OD of the culture at the sampling time. The slope of the resulting straight line is “Miller Units.”

The *lacZ* mRNA functional half-life was determined at an ambient temperature of 37°C as described (Pedersen et al., 2011). We induced the cultures for 5 or 10 min and then removed the inducer by filtration, washed the filter twice and re-suspended the cells in inducer free medium. Next, one milliliter samples were labeled at 20 s intervals with approximately 5 μCi carrier free [^35^S]-Methionine followed by a chase with more than 1,000-fold excess unlabeled methionine. After electrophoresis on a standard 7.5% SDS-acrylamide gel the protein bands were visualized on a phosphor image screen and quantified using the NIH-ImageJ software: imagej.nih.gov/ij/. Bands corresponding to the LacZ protein were normalized to the bands of the β/β′-RNA polymerase subunits. The labeling of these was not detectably influenced by the presence or absence of the *lac* inducer, 1 mM IPTG. The mRNA half-life was determined from the slopes in semi-logarithmic plots of the relative labeling of β-galactosidase to the radioactivity in the β and β′ subunits of the RNA polymerase vs. the time of labeling.

## Results

### Experimental results

As model for quantifying translation efficiency we use variants of the *lacZ* mRNA that differ in strength of the RBS, the distance of this sequence to the translational start site and with two sets of the first few codons. To differentiate the mechanisms that regulate the protein yield from each of these seven sequences we measured three different quantities: (1) the total protein yield, (2) the stability of the mRNA, and (3) the extent of premature transcription termination.

#### Protein yield: expression of β-galactosidase

The measured specific enzymatic activities from these *lacZ* variants normalized to the enzymatic activitiy measured from *lacZ*-wt are shown in Table 2, column A. We compared the specific enzymatic activity of the LacZ-rq enzyme to that of the wt enzyme and found it to be 85% of that of the wt enzyme. This was done by calibrating the enzymatic activity of the two enzymes to their actual amount as measured by the [^35^S] methionine labeled β-galactosidase bands in steady state of induction (data not shown). Table 2, column A shows the normalized protein yield corrected by this factor.

**Table 2:** **Expression of Protein and mRNA from the ***lacZ*** variants**.

**SD seq**.	**Coding seq**.	**A**	**B**	**C**	**D**	**E**	**F**	**G**
		**Relative amount β-gal protein**	**mRNA stability *t*_½_(s)**	**Relative β-gal prot. per mRNA**	**Fraction of successful transcription**	**β-gal protein per corrected mRNA**	**Time between initiation *t*_between_(s)**	**The first ribosome initiation time *t*_first_(s)**
sd5	Rq	0.0070 ± 0.0012	TS (25)	(0.033 ± 0.005)	0.16 ± 0.02	(0.21 ± 0.05)	(10.5 ± 2.5)	(9.5 ± 2.5)
sd5	WT	0.27 ± 0.05	64 ± 0.6	0.49 ± 0.10	0.86 ± 0.19	0.57 ± 0.24	3.9 ± 1.6	3.9 ± 1.6
SD8	Rq	0.28 ± 0.03	73	0.45 ± 0.05	0.98 ± 0.04	0.46 ± 0.07	4.8 ± 0.7	3.8 ± 0.7
SD8	WT	0.92 ± 0.11	117 ± 0.5	0.91 ± 0.11	1.12 ± 0.11	0.81 ± 0.17	2.7 ± 0.6	1.7 ± 0.6
SD12	Rq	0.061 ± 0.01	28	0.25 ± 0.04	0.67 ± 0.17	0.37 ± 0.15	5.9 ± 2.4	4.9 ± 2.4
SD12	WT	0.53 ± 0.10	108 ± 7.6	0.57 ± 0.15	1.05 ± 0.17	0.54 ± 0.24	4.1 ± 1.8	3.1 ± 1.8
WT	WT	=1	=116	=1	=1	1	=2.2	1.2

*Column A, Amounts of LacZ protein normalized to LacZ protein from wt lacZ. LacZ expression in the wt was 3,353 ± 245 Miller Units. The specific activity of LacZ in the variants with the rq coding sequence is approx. 85% of that for the wt enzyme. This correction was taken into account in the presented number. The values are obtained from average of at least 5 measurements, and the standard errors are shown. Column B, Half-life of the lacZ mRNA. TS: Too short to be measured. We use 25 s here in our further calculations, which the longest time where we estimate to be unable to measure the half-life due to the filtration, washing and resuspension procedure. This value, 25 s, and the values derived from it are uncertain and in parentheses. The values with standard errors are average over two measurements. Column C, Protein expressed per mRNA, normalized to the wt value. This value assumes no premature transcription termination. The amount of protein from column B per mean-life of the mRNA (= half-life/ln2) and then normalized to the wt. The mean-life is the hypothetical mRNA life-time if all mRNA were used for the fixed number of translations that give the same expression level as exponential decay. Column D, Successful transcription in the lacZ gene relative to wt that is the enzyme activity of LacA_variant_/LacA_wt_. The values are obtained from average of at least 4 measurements, and the standard errors are also shown. Column E, Column D corrected for the prematurely terminated mRNA transcription. (Column C)/(Column D). Column F, Average time between each translation initiation t_between_, assuming that the wt has 2.2 s between each initiation (Mitarai et al., 2008; 2.2/column E). Column G, The first ribosome initiation time t_first_, obtained by subtracting the occlusion time (~ 1 s) from Column F*.

The results confirm that the protein expression is hugely influenced by both the SD, its distance to the start site and by changes of the early codons in agreement with the previous results (Ringquist et al., 1992).

#### mRNA stability

The production of proteins depends on the lifetime of functional mRNA, which in turn can be measured by recording how the capacity for protein synthesis decays after transcription is suddenly stopped. The protocol for the used pulse-chase experiment is described in the method section. The measured half-lives for the seven *lacZ* variants are given in Table 2, column B. One see that the about 4-fold variation in the mRNA half-life can explain part of the variation in the total protein yield. However, the measured β -galactosidase yields in column A differed more than mRNA half-lives. In column C, we highlight this difference by tabulating the relative protein yield per functional mRNA. The protein yield per functional mRNA in column C may be attributed to either a variation in the translational initiation frequency or a change in the frequency of successful transcriptions.

#### Premature transcription termination

Even though our experimental setup is aimed at quantifying differences in translation while keeping the promoter and the first 30 nucleotide untranslated region of the mRNA unchanged, one should not forget that total protein yield is proportional to the number of completed transcription events which may be reduced by transcription termination. One mechanism would be a premature transcription termination in *lacZ*, e.g., associated to a lack of coupling between the first ribosome and the RNA-polymerase.

The *lac* operon consists of the genes: *lacZ, lacY*, and *lacA* and the *lac* mRNA has been found to be cleaved intergenically between *lacZ* and *lacY-lacA* (Cannistraro et al., 1986; Li and Altman, 2004). The *lacZ* and *lacA* mRNA thus have independent half-lives (Petersen, 1987; Murakawa et al., 1991). Consequently, *lacA* expression will be unaffected by changes in *lacZ*, unless an event in *lacZ* terminated the RNA polymerase and in this way lowered transcription of *lacA*. This assay was developed as the classical assay for polarity: Termination by transcription termination factor Rho if the leading ribosome fail to contact the polymerase for instance if translation was terminated by an early stop codon (Beckwith, 1963; Newton et al., 1965; Richardson et al., 1975). Such premature termination event might also affect messengers with weak RBS if the leading ribosome in these would take too long to contact the polymerase because of the reduced initiation frequency.

To measure premature termination, we completed the *lac* operon in these *lacZ* variants by inserting an *Eco*RI-*Eco*RI fragment that contained the *lacYA* part of the *lac* operon. We next measured the LacA transacetylase activity in these *lacZ* variants. Table 2, column E show the fraction of successful transcription events relative to that of the wt. For the weakest SD sequence, sd5, 16 or 84% of the RNA polymerases terminate before reaching *lacA* depending on the *lacZ* coding sequence. Noticeably, the stronger SD sequences are less sensitive to premature termination. Note that the *lacA* expression in the variant with the strongest *lacZ* RBS (SD8-*lacZ-wt*) is higher than that of the wt *lacZ* operon, indicating that only approximately 71% of the wt *lacZ* transcription initiations will lead to full length transcripts (see later).

#### Variation of the translation initiation

The expression of β-galactosidase differs more than the combined effect of mRNA half-life and the premature transcription termination can explain. The remaining differences in protein yield are explained by variation in translation initiation as shown in Table 2 column E.

One now observe a translation initiation frequency that at most vary 5-fold among these seven *lacZ* variants. Notice that estimate of the *sd5-lacZ-rq* variant is uncertain because of the low stability of this mRNA. Previously, we estimated the translation initiation frequency of the wt *lacZ* to be 1/2.2 s (Mitarai et al., 2008). Using this estimate, the average time intervals between subsequent translation initiations in the *lacZ* variants are shown in Table 2, column F. As we estimated the occlusion time of the ribosome to be about 1 s. (Mitarai et al., 2008), we in column G explicitly list the translation initiation time of first ribosome to be 1 s faster than the later ribosomes. In **Figure 2** we plot the estimated fraction of successful transcripts (from Table 2, column D) as function of the average time for the first ribosome initiation (column G).

### Model

This paper revisits the relationship between the RBS and the protein yield, using the *lac* system in *E. coli* as a model system. This has been done previously, most noticeably in the papers by Ringquist et al. (1992) and Yarchuk et al. (1992). In Ringquist et al. the explanation of the protein yield per message was entirely focused on ribosome initiation frequency while implicitly assuming that number of mRNA molecules and their stability were constants that were not influenced by translation. In Yarchuk et al. (1992) a connection between the efficiency of the initiation region and mRNA stability and premature transcription termination was established, but because of the large sequence variation among the initiation regions used, no mathematical model describing the connectivity between these mechanisms was attempted. The present paper includes wt SD and wt coding sequences and combines the systematic variance of the SD strength from the Ringquist et al. paper with measurements of both mRNA stability, protein yield per mRNA and the number of completed mRNA molecules to arrive to a simple mathematical model for the interplay between these three mechanisms. Furthermore, our results indicate that most, if not all, synthesis of natural mRNA are substantially affected by premature transcription termination.

We analyze our measurements in terms of a model that is inspired by the one used by Ringquist et al. (1992). As in that paper, we assume that ribosome binding and subsequent initiation frequency are central steps in determining the translation initiation frequency. In addition, we take into account the fact that ribosomes that initiated translation will occlude the binding of subsequent ribosomes until they have moved one ribosome diameter. This is important because the time between initiations and the occlusion time are only approximately 2-fold different. We estimated previously that subsequent ribosomes will initiate at about 2 s intervals whereas it takes a ribosome about 1 s to translate the about 11 codons that would make space for binding of the next ribosome (Mitarai et al., 2008).

To simplify the presentation of our model we describe translation initiation as a 2-step process (and not the 3-step model used by Ringquist et al.). We assume that the ribosome binds to the RBS with a rate *k*_*1*_ and unbinds with a rate *k*_−*1*_. In other words, each time a ribosome binds, then it stays at the RBS for a time interval of average duration 1/*k*_−*1*_. During this time, the ribosome can transit to start translation with a rate *k*_*2*_, as illustrated in the insert of **Figure 3**. If the ribosome starts translation we assume that the ribosome is now committed, and will stay on the mRNA for as long as translation is possible. The average time for the first ribosome to start translation is then

$$\begin{array}{l} t_{\text{first}}=t_{\text{unbound}}+1/k_{\text{2}}=\left(1/k_{\text{1}}\right)\times \left(1+k_{-\text{1}}/k_{\text{2}}\right)+1/k_{\text{2}} \end{array}$$

Here the first term *t*_unbound_ is the average time where the ribosome is uncommitted/unbound, which is calculated by multiplying the time for binding 1/*k*_*1*_ with the average number of binding attempts before initiation wins over that unbinding (1 + *k*_−*1*_/ *k*_*2*_). The second term 1/*k*_*2*_ is the time it typically takes for a ribosome to start translating, given that it is bound at the RBS. This model is a simpler version of the 3-step model used by Ringquist et al. and could as well describe their data by rephrasing the above rate constants *k*_*2*_ and *k*_−*1*_ in terms of combinations of the additional rate constants that ware introduced by Ringquist et al. (1992).

The time before the first ribosome starts translating should be shorter than the time intervals between translation starts of the subsequent ribosomes, because the previous ribosome occlude binding of the subsequent ones for the average time, *t*_occlusion_, it takes a ribosome to clear the RBS. The *t*_occlusion_ was estimated to be 1 s (Mitarai et al., 2008). Thereby we expect that the time between subsequent ribosome initiations would be given by

$$\begin{array}{l} {\text{t}}_{\text{between}}=t_{\text{occlusion}}+\left(1/k_{\text{1}}\right)\times \left(1+k_{-\text{1}}/k_{\text{2}}\right)+1/k_{\text{2}}. \end{array}$$

This relation predicts that there is an upper limit for translation initiation; it cannot be faster that once per occlusion time or approximately once per sec. Also, it implies that when this limit is approached, the distance between subsequent ribosomes will be small which likely will cause ribosome collisions and queuing and also excluding any other factors from access to the mRNA.

**Figure 1:**
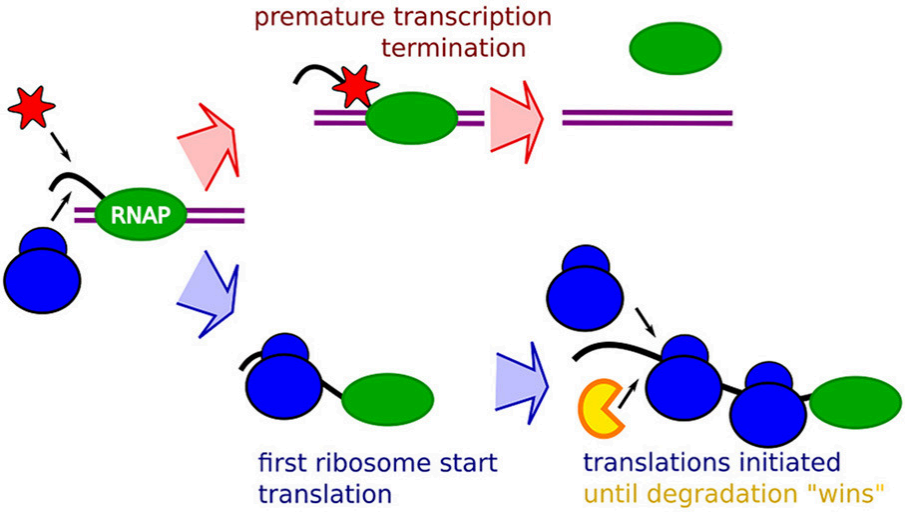
**Schematic description of the RBS occlusion model**. First, a RNA Polymerase (Green) on double strand DNA (purple) start transcribing a mRNA (black). The race between occlusion by the first ribosome (blue) and arrival of transcription terminating enzymes which may be the Rho factor (red) determines the extent of the premature termination. If the first ribosome successfully starts translation it occludes the transcription terminating enzyme, and the mRNA stability will subsequently be affected by occlusion of RBS preventing binding of the mRNA degrading enzymes (yellow) around the RBS by initiating ribosomes.

The notion that occlusion will have an increasing relative effect with faster ribosome initiation rates suggests that all our data could be explained in terms of occlusion by ribosome, as schematically described in Figure 1. The premature termination is coupled to the absence of occlusion, allowing binding of transcription termination factors, possibly transcription termination factor Rho, Figure 1 top. The mRNA stability will then be explained by occlusion that prevents binding of the degrading enzymes. An mRNA with lower translation initiation rate would then at the same time be “hit” by an increased premature termination, and a shorter life-time.

In the simplest formulation of such a model, we will assume that both mRNA stability and premature termination is determined in the beginning of the mRNA. This is motivated by the observation that only 0.4% of peptide are tagged by tmRNA rescue mechanism (Moore and Sauer, 2005). If there were significant mRNA cleavages between translating ribosomes, the use of the tmRNA rescue mechanism should be larger, because the estimate of the number of peptides produced per mRNA for the wt *lac* mRNA is approximately 75, based on the wt *lac* mRNA mean life (116/ln2 s) and the initiation frequency (1 per 2.2 s). This suggests that mRNA degradation starts early on the mRNA, so it does not interfere with the translating ribosomes (Pedersen et al., 2011). Premature termination by Rho is also believed to terminate mRNA synthesis if the mRNA is not protected by the contact between the first translating ribosome and the RNA polymerase (e.g., Newton et al., 1965).

Within this framework we will first assume that premature termination can act in the time interval before the first ribosome has reached the elongating RNA polymerase. This time of exposure to termination factors is proportional to

$$\begin{array}{l} t_{\text{exposure to termination}}=\text{constant}\times t_{\text{first}}\\ =\text{constant}\times \;\left(t_{\text{between}}-t_{\text{occlusion}}\right) \end{array}$$

The “constant” depends on the difference in speed between first ribosome and the RNAP when it is transcribing without the help of an associated ribosome. In this simple formulation, the probability that the mRNA survives this termination is

$$\begin{array}{l} S=\text{exp}\left(-k\times t_{\text{exposure to termination}}\right)=\text{exp}\left(-t_{\text{first}}/7s\right) \end{array}$$

**Figure 2:**
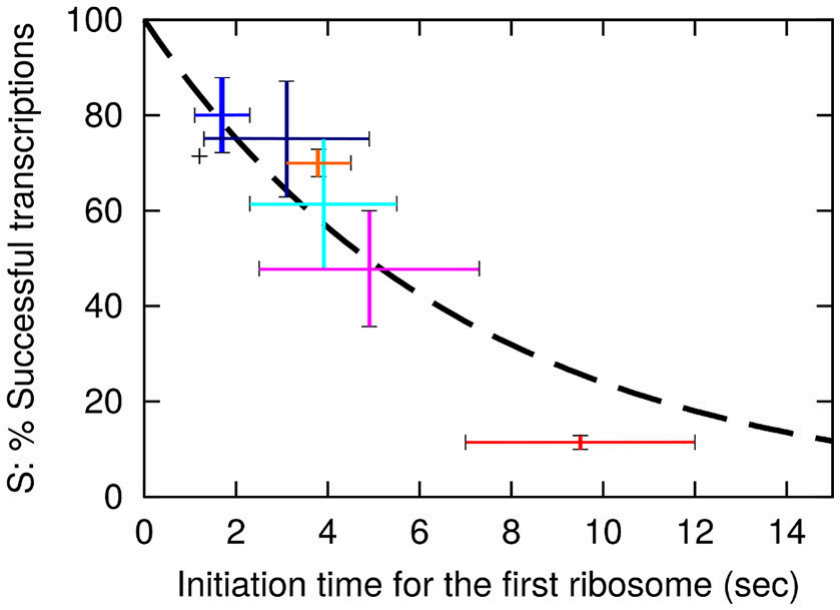
**The probability, S for a transcription event to be successful is plotted as a function of the initiation time ***t***_**first**_ of the first ribosome**. The dashed line shows the fit *S* = exp(−*t*_first_/(7 *s*)) with *S* value for the wt of 0.71. The vertical axis is therefore the values in Table 2 column E multiplied by 0.71. Error bars for the data are based on the SEM in the LacA transacetylase activity measurement, where at least 3 independent measurements were made. Black: wt-*lacZ*-wt, red: sd5-*lacZ*-rq, cyan: sd5-*lacZ*-wt, orange: SD8-*lacZ*-rq, blue: SD8-*lacZ*-wt, magenta: SD12-*lacZ*-rq, navy: SD12-*lacZ*-wt.

Here *k* is the rate for premature termination factors to engage. The final fit that use the exponential decay with decay time of approximately 7 s is shown as a dashed line in Figure 2.

Remarkably, this fit predicts that even *lacZ*-wt sequence is exposed to approximately 29% premature termination, while the SD8-*lacZ*-wt sequence is still exposed to 20% premature termination. Further, the above equation is simplified, in the sense that we assume that the time between initiation of subsequent translations is fixed, corresponding to a near deterministic sequence events for each initiation. If each translation start is primarily governed by a single rate limiting event, the functional fit becomes a hyperbola, *S* = 1/(1 + *t*_first_/τ), and the best fit for the constant τ predicts a premature termination of wt *lacZ* mRNA of about 33% (analysis not shown).

In our occlusion based model, the stability of the mRNA is also determined by translation initiation, as the mRNA is exposed to the degradation during the times when the ribosome is not bound, i.e., during the time *t*_unbound_ between the subsequent ribosome initiation. In other words, we can express the degradation rate being proportional to the probability that the translation start site is free from a ribosome

$$\begin{array}{l} P_{\text{free}}=t_{\text{unbound}}/t_{\text{between}}. \end{array}$$

Table 2 give us *t*_between_ for each construct, but does not give us direct information about individual rates, *k*_*1*_, *k*_−*1*_, and *k*_*2*_, which is needed to calculate *t*_unbound_. The ribosome on-rate *k*_*1*_ should be independent of RBS, and substantially faster than the shortest of our *t*_between_ allows for. For simplicity, we fix its value to *k*_*1*_ = 10/s. We then assume that, when RBS of a mRNA is not occluded by a ribosome, mRNA degradation enzymes act on the mRNA with the same rate “η.”

**Figure 3:**
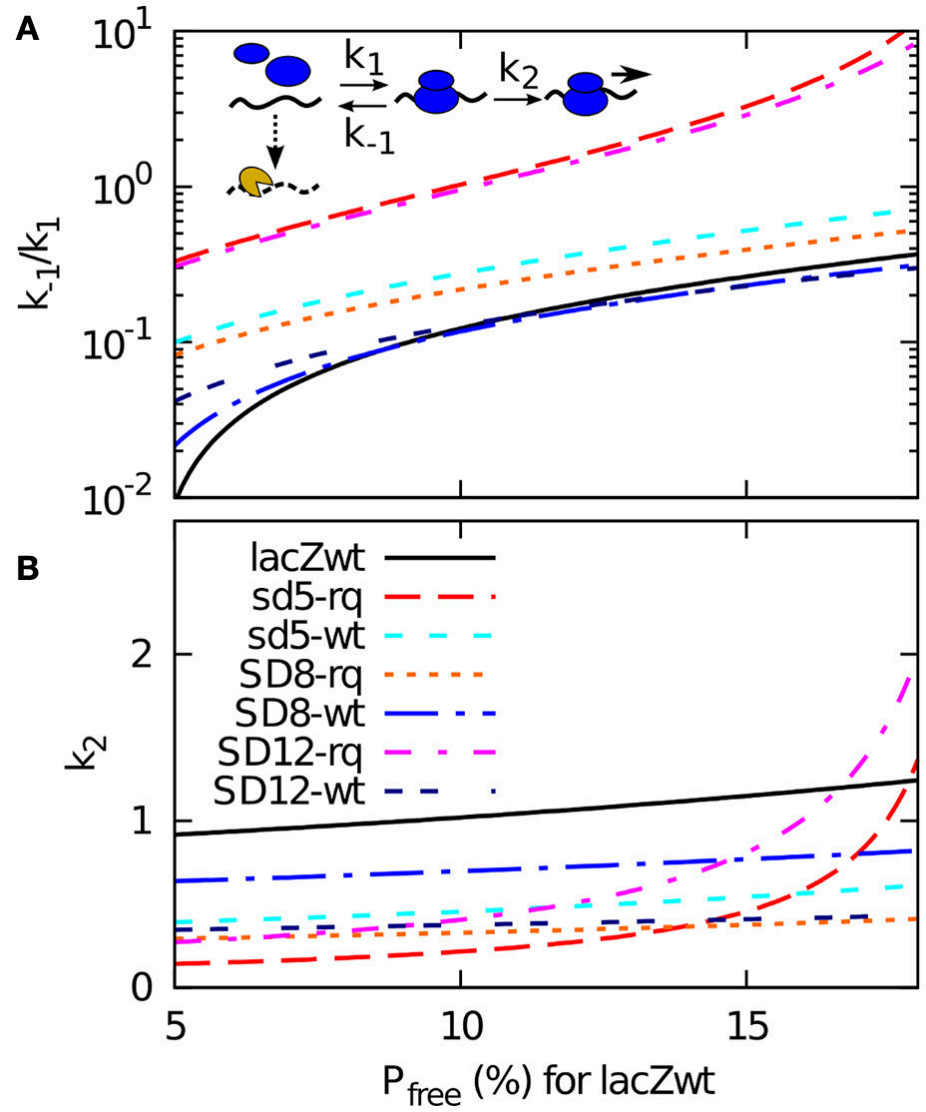
**A 2-step model of translation initiation and mRNA degradation**. Insert: we assume that ribosome (blue) binds to RBS on mRNA at a rate *k*_*1*_, dissociate at a rate *k*_−*1*_, or start initiation at a rate *k*_*2*_. After initiation, it will take occlusion time *t*_occlusion_ of 1 s before the next ribosome can bind. The mRNA degrading enzyme complex is shown in yellow, and it can attack at a constant rate η to the mRNA if the RBS is free. The mRNA degradation rate is assumed to be η × *P*_free_, where *P*_free_ is the probability that a RBS is free from ribosome. Experimental data gives the time between the initiation *t*_between_, and to calcucate *P*_free_ we also need the effective dissociation constant (*k*_−*1*_/*k*_*1*_) and the effective firing rate *k*_*2*_. From the relations given in the main text, and knowing that the degradation rate is the inverse of the mean life time *t*_*1/2*_/log(2), we obtain *t*_unbound_ = *t*_between_ × (log(2)/*t*_*1/2*_)/η*, k*_*2*_ = 1/(*t*_between_ − *t*_between_ − *t*_unbound_), and (*k*_−*1*_/*k*_*1*_) = *k*_*2*_ × (*t*_unbound_ − 1/ *k*_*1*_). These relations enable us to plot (*k*_−*1*_/*k*_*1*_) and *k*_*2*_ for all the sequences using the measured values of *t*_*1/2*_ and *t*_between_ for a given η. In **(A,B)**, we plot them as a function of the probability *P*_free_ that RBS is free for wild type sequence. This probability can be converted to the rate of degradation for naked mRNA η by using wt mRNA's half-life 116 s as *P*_free_ = log(2)/116 s/η.

The lifetime of each mRNA is now fitted by the mRNA degradation rate *P*_free_ × η. Given that the wild type mRNA has a half-life of approximately 116 s (Petersen, 1987), each choice of η correspond to a particular value of *t*_unbound_ (hence *P*_free_) for the *wt lacZ*. Or reversely, we can determine η from assuming a fraction of time that the wild type sequence is exposed to the degrading enzymes. In Figure 3 we use this way to present our data, where a *P*_free_ being 5 % for *wt* RBS site correspond to a much higher η than the case where the *wt* RBS is exposed 15% of the time. For each value of this exposure (and thereby of η) our model provides a value of the dissociation constant *k*_−*1*_/*k*_*1*_ and the firing rate of a bound ribosome *k*_*2*_ that match the measured half-life (Table 2 column B) and the measured translation initiation frequency (Table 2, column F).

While all the curves presented in Figure 3 represent possible fits to our data, the figure also provide some constraints on plausible parameters. Notice for example that some of the very weak start sites such as SD12-lacZ-rq or sd5-lacZ-rq have high values of both firing rate *k*_*2*_ and of dissociation constant *k*_−*1*_/*k*_*1*_ for larger *P*_free_ (hence smaller η). This is because, to fit their short life-times, *t*_unbound_ for these sequences needs to become large. It is not plausible that the relatively weak start sites of SD12-lacZ- rq or sd5-lacZ-rq should have a faster *k*_*2*_ rate than the other sequences, which in turn suggest that the *wt* RBS is exposed to degradation for less than 10% of the time. For this relatively high activity of the degradation the overall set of suggested parameters are less extreme.

## Discussion

We propose a model for ribosome initiation and the resulting protein yield. Our model extends the Ringquist et al. model for translation initiation to also take into account ribosome occlusion, which we suggest, influence both translation initiation, the premature transcription and the functional stability of the mRNA. More recently, Richards et al. (2012) observed a similar effect for the chemical half-life of mRNA based on experiments where the average ribosome distance was varied by altering the RBS. This work also indicated a role for the translation machinery in influencing the mRNA stability. Our model was supported by experiments that allowed us to quantify each of the factors that determine the overall protein yield by systematically altering RBS. Overall we saw that a 120-fold change in protein yield could be explained by a product of three different and roughly equally contributing factors: Translation initiation rate, premature transcription termination and mRNA stability. Three factors that can be consistently explained by assuming that the parameters of the initiation rate are the prime determinants, that in turn influence both of the two subsequent factors.

We speculate that our finding from Figure 2, that the strength of the RBS influences the mRNA level in a cell, may provide a more versatile regulation compared to a cell that relies on promoter control only. This figure suggest that the mRNA level can be directly modulated by the relative speeds of the ribosome and the RNA polymerase. The mRNA level therefore may be regulated by external conditions that result in altered nucleotide triphosphate concentrations and/or tRNA charging levels. The codon usage in the early coding region in general has a lower Codon Adaptation index (Sharp and Li, 1987; Bulmer, 1988) which in all likelihood means that the ribosomes translating this part of the mRNA are unsaturated with substrate. The speed of translating ribosome should therefore be able to respond to changes in external conditions.

Finally, our suggested role of a premature transcription in fine-tuning mRNA transcription might explain at least part of the discrepancy between the observed total number of RNA polymerases and the number estimated for synthesis of mRNA and stable RNA. The seemingly “inactive RNA polymerases” are about twice that of the “active polymerases” (Bremer and Dennis, 1996). As shown in Figure 2 even an mRNA with an extremely good SD such as SD8-*lacZ-wt* is still subject to approximately 20% premature termination. The premature transcription termination of most natural mRNA therefore can be expected to be substantially higher. Compared to the regular promotor control a premature transcription termination mechanism may respond better to external signals and might therefore be advantageous for the cell.

## Author contributions

ME and SP performed and designed experiments; NM and KS performed the modeling; NM, KS, ME, and SP wrote MS.

### Conflict of interest statement

The authors declare that the research was conducted in the absence of any commercial or financial relationships that could be construed as a potential conflict of interest. The reviewer CB and handling Editor declared their shared affiliation, and the handling Editor states that the process nevertheless met the standards of a fair and objective review.
